# Clinical Implementation of Predictive Models Embedded within Electronic Health Record Systems: A Systematic Review

**DOI:** 10.3390/informatics7030025

**Published:** 2020-07-25

**Authors:** Terrence C. Lee, Neil U. Shah, Alyssa Haack, Sally L. Baxter

**Affiliations:** 1 Viterbi Family Department of Ophthalmology and Shiley Eye Institute, University of California San Diego, La Jolla, CA 92093, USA; 2 Division of Biomedical Informatics, Department of Medicine, University of California San Diego, La Jolla, CA 92093, USA

**Keywords:** electronic health records, predictive models, predictive analytics, risk prediction, clinical prediction model, precision medicine, clinical informatics, clinical decision support, artificial intelligence

## Abstract

Predictive analytics using electronic health record (EHR) data have rapidly advanced over the last decade. While model performance metrics have improved considerably, best practices for implementing predictive models into clinical settings for point-of-care risk stratification are still evolving. Here, we conducted a systematic review of articles describing predictive models integrated into EHR systems and implemented in clinical practice. We conducted an exhaustive database search and extracted data encompassing multiple facets of implementation. We assessed study quality and level of evidence. We obtained an initial 3393 articles for screening, from which a final set of 44 articles was included for data extraction and analysis. The most common clinical domains of implemented predictive models were related to thrombotic disorders/anticoagulation (25%) and sepsis (16%). The majority of studies were conducted in inpatient academic settings. Implementation challenges included alert fatigue, lack of training, and increased work burden on the care team. Of 32 studies that reported effects on clinical outcomes, 22 (69%) demonstrated improvement after model implementation. Overall, EHR-based predictive models offer promising results for improving clinical outcomes, although several gaps in the literature remain, and most study designs were observational. Future studies using randomized controlled trials may help improve the generalizability of findings.

## 1. Introduction

Predictive analytics is a rapidly expanding area of health care [1]. With widespread electronic health record (EHR) adoption [2–4], vast quantities of clinical data are available. EHR data have been employed to develop predictive models in a wide range of clinical applications, such as predicting major post-surgical complications [5–7], sepsis [8], readmission [9–11], heart failure [12], substance abuse [13], and death [9,14,15]. Computational advancements have enabled machine learning techniques to effectively use EHR data for medical diagnosis [16–18]. The stated promise of these predictive models is to improve identification and risk stratification of patients, thereby facilitating targeted interventions to improve patient outcomes. Embedding these models within EHR systems as components of clinical decision support (CDS) interventions may allow real-time risk prediction. As stated by Agrawal and colleagues [19], this “prediction technology” is core to the anticipated applications of artificial intelligence (AI) for improving health care.

However, the implementation of predictive models into EHR systems for clinical practice is not straightforward. This is an emerging area of investigation where best practices are not yet well established. Although CDS interventions have been employed in EHR systems for many years, the emergence of more advanced computational models, such as the use of machine learning approaches, presents some key challenges and unique considerations. Described in previously published frameworks, such as those by Shaw et al. [20] and He et al. [21], these include explainability and transparency of algorithms, computational resources, scalability, data standardization, and integration into clinical workflows as meaningful decision support.

Although these prior articles have outlined key implementation issues around EHR-based predictive models, they examined only a limited set of use cases. A systematic review by Goldstein et al. [17] examined risk prediction models utilizing EHR data, but focused on model development and validation rather than implementation in clinical settings. Another systematic review by Kruse et al. [18] regarding the challenges of opportunities of big data in health care also included discussion of predictive models, but its search encompassed articles only up to 2015. The rapid growth of this field in recent years offered us an opportunity to provide an updated review, with a focus on implementation issues.

Here, we engaged in a systematic review of EHR-based models that have been implemented in clinical practice using a rigorous search methodology. The objectives of our study were to review published peer-reviewed articles describing predictive models that have been implemented in real-world clinical practice, summarize their findings, and highlight lessons learned to further inform the literature regarding implementation of these emerging technologies in health care settings.

## 2. Materials and Methods

We utilized the Preferred Reporting Items for Systematic Reviews and Meta-Analyses (PRISMA) Statement for reporting our systematic review [22,23]. The PRISMA reporting checklist can be found in Supplementary Table S1.

### 2.1. Eligibility Criteria

We limited our systematic review to peer-reviewed journal articles published in English between 1 January 2010 and 31 July 2019 with available full text. We chose 2010 as the start date given the passage of the Health Information Technology for Economic and Clinical Health (HITECH) Act in 2009 [24], which was aimed at promoting adoption and meaningful use of health information technology, including EHRs. Prior to the HITECH Act, EHR adoption was relatively limited and implementation of EHR-based predictive models was uncommon. Furthermore, we wanted to focus on predictive models implemented within the last decade to provide a more recent perspective.

Our primary eligibility criteria focused on identifying articles with the following: description of a model predicting a clinical outcome (e.g., not financial outcomes), use of EHR data for modeling, automated data extraction for modeling (e.g., not manual data entry or manual data calculation by providers), integration of the model into the EHR system, and implementation into clinical use. This last criterion was critical given the emphasis of our review on implementation, rather than on model development, training, or validation. We did not restrict studies to specific types of models—for instance, models using linear regression, logistic regression, random forests, and various neural network architectures were all eligible. However, our definition of “model” did require that there be some sort of mathematical calculation involving predictors based on EHR data. Therefore, CDS interventions with simply rule-based or criteria-based logic were not included. This allowed us to specifically focus on predictive analytics. To provide a broad overview of implemented EHR-based predictive models, we did not have any restrictions on clinical domains, patient populations, or study designs.

### 2.2. Information Sources and Searches

We conducted searches in the following six databases to reflect the interdisciplinary approach in predictive modeling: PubMed, Web of Science, Embase, Cochrane Library, CINAHL, and Business Source Complete. For our search strategy, we identified three primary concepts related to our study question: (1) electronic health records; (2) predictive models; and (3) implementation. For the first concept, we included terms related to electronic health records, electronic medical records, and computerized patient records. For the second concept, we included terms related to predictive models, algorithms, artificial intelligence, machine learning, informatics, risk prediction, statistical models, and clinical decision support. For the final concept, we included terms related to implementation, implementation science, and real-world or applied/practical trials. Within our searches, we employed structured vocabulary terms (if applicable to the given database), synonyms, and free-text title/abstract searches. We included filters for English-language and full-text articles only that were published within the specified date range. We included truncation and wildcards to allow the search to include articles with minor spelling variations to the indicated search terms. The search strategy was iteratively refined with the assistance of a university librarian with extensive experience in systematic review methodology. To illustrate, the detailed PubMed search strategy which includes all search terms can be found in Appendix A. In addition to database searches, we also identified potential articles via reviews of bibliographies in articles identified from the database searches, expert recommendations, and manual/hand searching.

### 2.3. Study Selection

The articles resulting from all search methods described above were collated and screened. First, we removed any duplicates and any articles that had been retracted. The remaining articles underwent title/abstract review by two independent reviewers (T.C.L. and N.U.S.). Discrepancies were resolved by a third reviewer (S.L.B.) to generate a list of articles for full-text review. Four reviewers (T.C.L., N.U.S., A.H., and S.L.B.) conducted full-text article review, each beginning with an initial review of a portion of the studies for eligibility. Studies marked as not meeting eligibility criteria were reviewed by two reviewers (S.L.B. and T.C.L.) for confirmation. This generated the final set of full-text articles for data extraction and inclusion for qualitative analysis.

### 2.4. Data Collection and Quality Assessment

Full-text articles were divided among four reviewers (T.C.L., N.U.S., A.H., and S.L.B.) for data extraction. A spreadsheet was used to standardize data collection. The following items were extracted: publication year, first author’s name, title, journal, location of study (city, state, country), health system setting (inpatient/outpatient, academic/community, number of clinical sites), clinical outcome for predictive model, study design (e.g., randomized trial, pre–post analysis), patient population, control/comparison group (if applicable), study period (dates), sample size, EHR vendor, intended users of model (e.g., physicians, nurses, care coordinators), method of modeling (e.g., regression-based methods vs. non-regression based methods that tend to be more computationally intensive such as random forests, gradient-boosted trees, or neural networks), custom model developed by study authors or “off-the-shelf” model from EHR vendor or other source, method of risk score presentation to end users (e.g., dashboard, alert or best practice advisory; interruptive vs. non-interruptive), mention of alert fatigue, stand-alone intervention vs. component of broader intervention, measured effect(s) of predictive model, study quality rating, overall change in clinical outcomes, and key insights regarding model implementation. The risk of bias in individual studies was guided by the Downs and Black checklist [25], which reviewers referred to when making quality assessments. Due to the heterogeneity of clinical domains and studies included in the review, qualitative summaries of quality assessments were made in lieu of quantitative comparisons. Risk of bias across studies was mitigated via searching multiple databases, which increased the number of available records and expanded the search across multiple disciplines.

### 2.5. Synthesis and Analysis of Results

Because the review included studies regarding a range of different clinical outcomes, we did not calculate quantitative summary measures such as risk ratios or conduct meta-analyses. We performed a qualitative synthesis of the included studies. All reviewers convened as a group to identify key findings based on the previously described data extraction. Each reviewer then returned to their assigned full-text articles to identify and categorize relevant articles by key themes (e.g., abbreviated study design, custom model vs. “off-the-shelf” model, interruptive vs. non-interruptive vs. not reported alerts, mention of alert fatigue, and overall change in outcome). Each reviewer was then assigned a random sample of articles reviewed by others to verify coding. Any discrepancies were resolved by consensus from the entire group of reviewers.

## 3. Results

### 3.1. Study Selection

**Figure 1.**
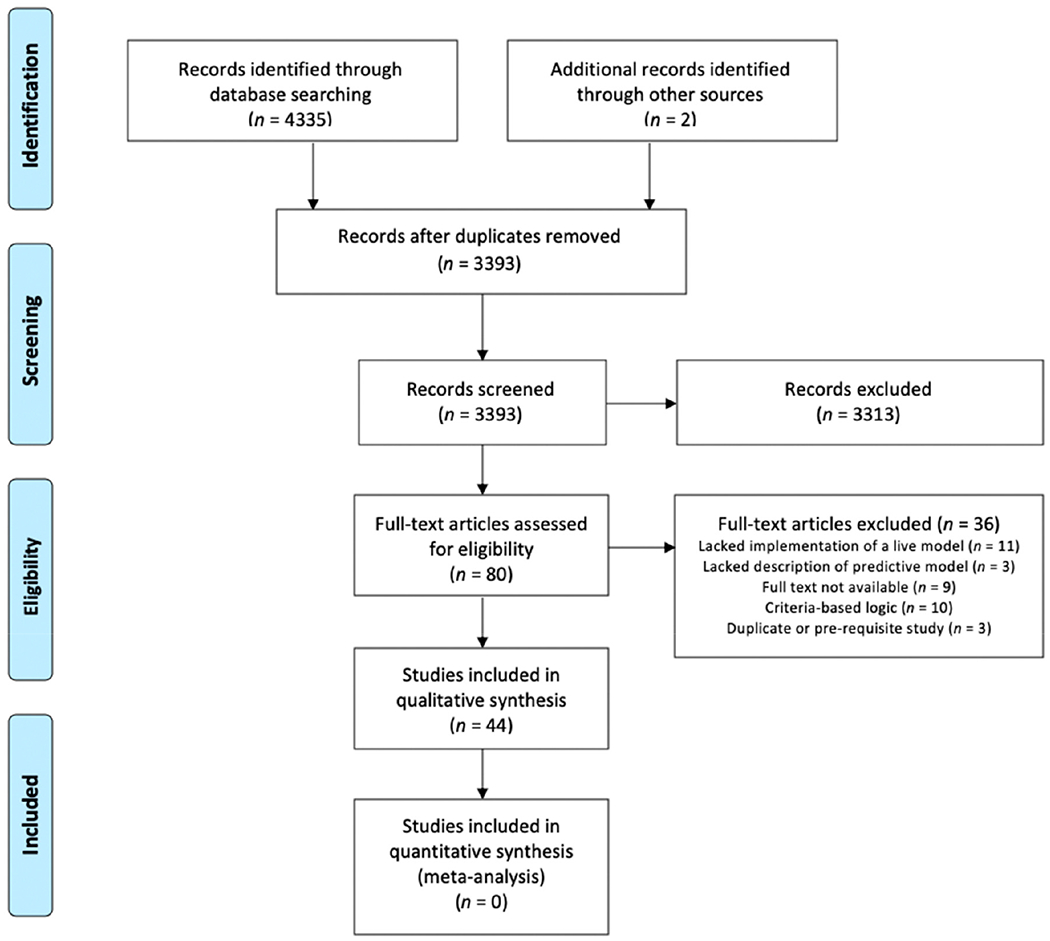
PRISMA flow diagram describing the study selection process.

Based on our aforementioned search strategies, we obtained an initial set of 3393 articles for screening. Distribution of articles bye database is depicted in Supplementary Figure S1. We conducted a title/abstract review and excluded studies based on the established eligibility criteria, resulting in 80 articles for full-text review. After full-text review, a final set of 44 articles was included for data extraction and qualitative analysis. Figure 1 depicts a detailed PRISMA flow diagram describing study selection.

We excluded articles during title/abstract review and during full-text review for a myriad of reasons. We excluded studies that were only conference or meeting abstracts without full-text journal articles available. Our intended focus was on implementation, so we excluded studies focused solely on model development (even if there was an external validation cohort) if there was no evidence of real-world clinical implementation. We excluded studies describing prescribing error notifications, dosing guides/recommendations, and studies focused on antimicrobial stewardship, as these generally used rule-based or criteria-based logic, whereas we were interested in predictive models requiring some extent of mathematical computation of risk. We also excluded studies focused on computerized physician order entry (CPOE) using order sets without risk prediction, studies describing a model prototype but no implementation, studies describing work related to health IT infrastructure but not a specific model, studies involving external software applications (e.g., mobile health devices) or web-based tools that did not have any links back into EHR systems, studies involving manual data collection and calculation, studies describing tools to assist with guideline adherence but without any risk calculation, studies involving tools based on rule- or criteria-based logic without any modeling or calculation, studies outlining rationale and design of proposed clinical trials but without actual results reporting, studies describing alerts of recently placed similar orders or of general reminders but without modeling or risk prediction, and studies asking physicians to manually document their personal risk assessment instead of a prediction based on EHR data modeling. In addition, we were focused on medical settings for humans, so we excluded studies from dental and veterinary settings.

### 3.2. Study Characteristics: Study Settings, Study Design, and Clinical Domains

**Figure 2.**
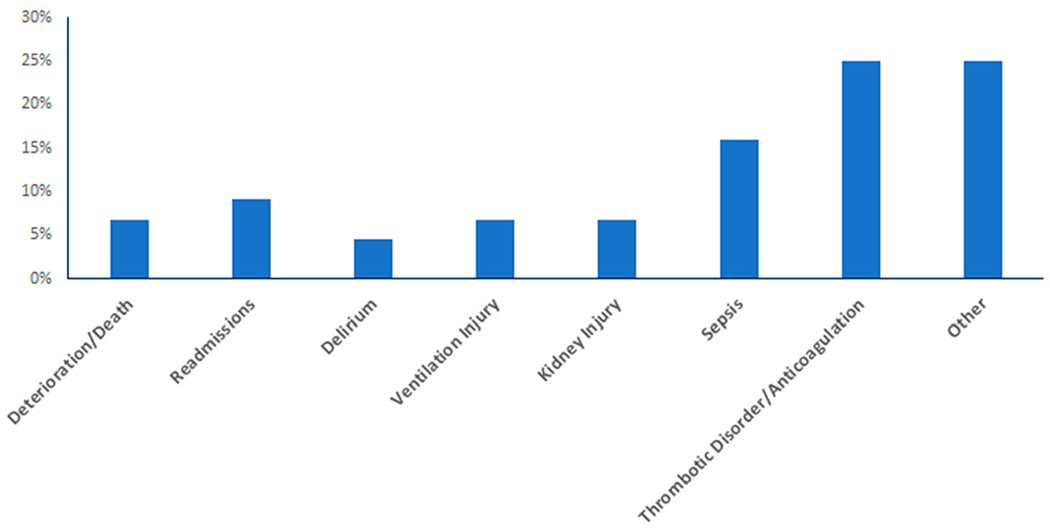
Distribution of studies regarding implementation of EHR-based predictive models based on primary clinical outcome.

Table 1 provides an overview of the studies that were included in the review. The most common clinical domains for predictive models embedded ln EHRs and implemented in clinical practice were models related to thrombotic disorders/anticoagulation (11/44 studies, 25.0%) and sepsis (7/44,15.9%) (Figure 2). Other domains included kidney injury, ventilation injury, delirium, readmissions, and deterioration/death. Tire remaining studies (grouped together as “other” in Figure 2) consisted of miscellaneous clinical entities, such as back pain, pressure ulcers, hypertension, perioperative risk, and triage time, among others. The majority (36/44, 81.8%) of studies were conducted in the United States.

The majority (28/44, 63.6%) of studies were centered on inpatient populations, while 16/44 (36.4%) studies concerned clinical predictions in outpatient settings. Additionally, 21 (47.8%) studies were conducted in an academic setting, 7 (15.9%) studies were conducted in a community setting, and 3 (6.8%) studies were conducted in a mix of both academic and community settings. The remaining 13 (29.5%) studies did not report a study setting that could be clearly classified as “academic” or “community”.

### 3.3. Predictive Models

Of the 44 studies, 30 (68.2%) utilized custom models, defined as a model developed by the authors or a previously validated model modified by authors to meet site-specific implementation needs, while 14 (31.8%) studies utilized “off-the-shelf” models, defined as a model previously developed and validated and implemented without site-specific modifications. Out of the 30 studies describing custom models, 17 (56.7%) were based on regression modeling, while 3 (10%) were developed using machine learning or deep learning. The remaining 10/30 studies (33.3%) did not report specific modeling methods.

### 3.4. Integration into EHR Clinical Decision Support Tools and Implementation Challenges

**Figure 3.**
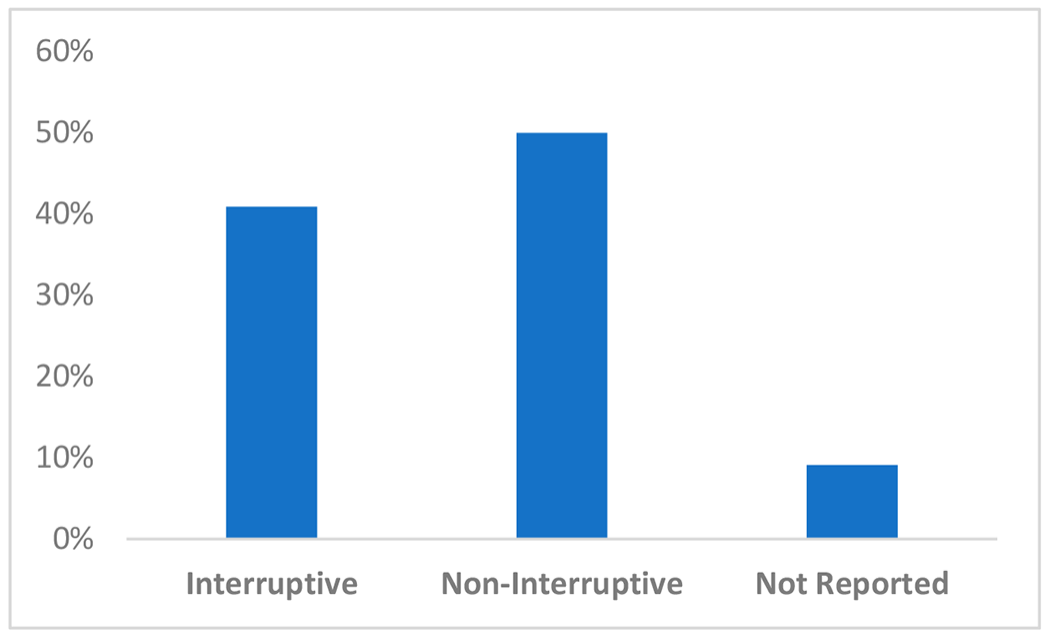
Distribution of risk score presentation from predictive models within electronic health record (EHR) systems when classified as interruptive or non-interruptive.

We categorized studies based on whether interruptive alerts or non-interruptive alerts (e.g., dashboards) were used during implementation to present risk scores or results of the predictive models to end users. Half of the studies (22/44, 50.0%) reported using non-interruptive alerts at intervention sites (22/44,50%), while 18/44 studies (40.9%) incorporated interruptive alerts. Four (9.1%) studies either did not report the risk score presentation to end users or were unable to be classified as interruptive or non-interruptive (Figure 3).

One common theme we observed was the mention of alert fatigue, defined as an inadequate response to a clinical decision support alert due to the frequency and increased burden on health care providers [70]. Alert fatigue was discussed in 14 of the 44 studies (31.8%). Of these 14 studies in which alert fatigue was mentioned, 11 (78.6%) utilized interruptive alerts, defined as alerts that either required action to dismiss the alert or alerts that significantly diverted the provider’s attention (e.g., text paging, calls). Three (21.4%) utilized non-interruptive alerts, defined as passive displays and notifications (e.g., sidebars, dashboards, or floating windows) that did not require specific action or divert the provider’s attention. However, six of these studies mentioned alert fatigue briefly but did not elaborate with any significant detail. We selected the remaining eight of these studies to further examine the specific description of risk score presentation and extracted representative quotations to gain insight on the role of alert fatigue in predictive model implementation (Table 2).

Other than alert fatigue, other implementation challenges were also noted. Several studies reported intrinsic challenges, which we defined as issues that arose from model design and development. These challenges include limitations in the predictive model’s user functionality, overconsumption of resources, and requiring access to costly data [36,47,53]. Arts et al. also cited non-interruptive risk score presentation as a reason for low usage, thus affecting the performance of the predictive model [47]. Other studies noted that some barriers of implementation may be linked to the preliminary development of predictive models, such as in mapping the correct EHR fields for the desired data elements [55].

Several studies also reported extrinsic challenges, which we defined as issues that were introduced in the clinical setting (e.g., disruption of workflow). Issues such as lack of training or lack of familiarity by rotating trainees, increased work burden on the care team, and the introduction of extra work discouraged use of several predictive models [28,43,55]. One predictive model that risk stratified Pediatric Appendicitis Scores (PAS) was deemed irrelevant, as clinicians believed that PAS guidelines could be easily memorized and thus did not require decision support [43]. Another extrinsic challenge included “evolving clinical profiles,” as described by Hao et al. [39] in the performance of a 30 day readmission risk assessment tool.

Of note, Khoong et al. [68] illustrated a theory-based strategy to encourage provider uptake of predictive models. The capability, opportunity, motivation, behavior framework (COM-B) asserts that capability, opportunity, and motivation are essential conditions that impact behavior. In their study, Khoong et al. [68] addressed implementation barriers by educating providers about the model (capability barriers), fitting the model to physician workflow by streamlining patient education and orders (opportunity barrier), and providing incentives and reminders to encourage use of the model (motivation barrier).

### 3.5. Impacts on Clinical Outcomes

We evaluated the included studies for results describing whether the implementation of a predictive model yielded improved clinical outcomes. Of the 44 studies evaluated, 12/44 (27.3%) did not include an evaluation of clinical outcomes. Often, these focused on performance metrics (e.g., positive predictive value, negative predictive value) of the model itself rather than on effects on clinical outcomes. For example, Moon et al. [59] reported high levels of predictive validity for an automated delirium risk assessment system; however, the authors did not report changes in clinically diagnosed delirium. Other studies that did not evaluate clinical outcomes showed changes in other clinical aspects such as improved time savings [55].

**Figure 4.**
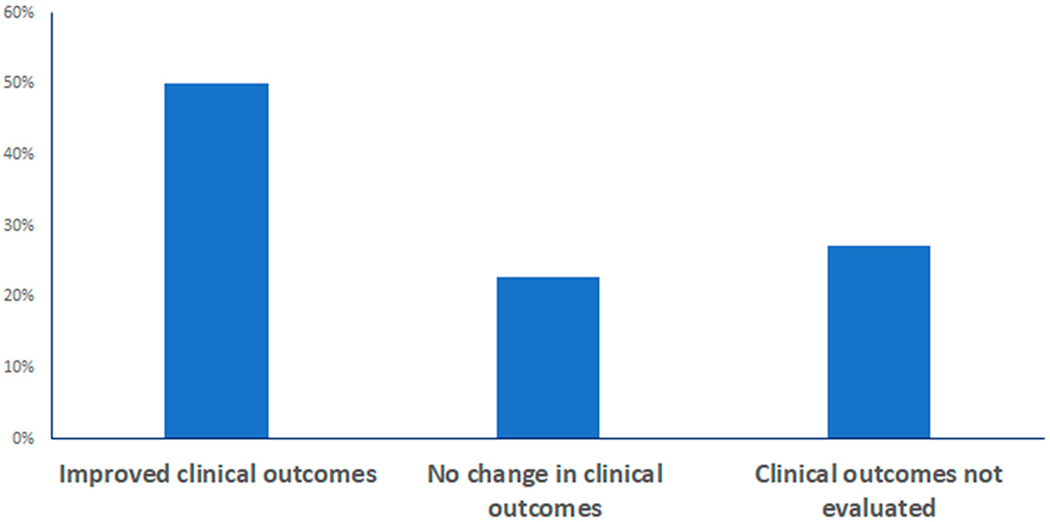
Distribution of studies regarding effects on clinical outcomes after implementation of EHR-based predictive models.

Twenty-two (50.0%) studies evaluated clinical outcomes and showed an improvement in clinical outcomes, while 10 (22.7%) evaluated clinical outcomes and showed no improvement or change (Figure 4). Clinical outcomes were not evaluated in 12 (27.3%) studies. Similar to studies that did not evaluate clinical outcomes at all, studies that showed no improvements in clinical outcomes often reported secondary benefits. For instance, Oh et al. [35] reported no changes in the incidence of delirium with an automatic delirium prediction system; however, a significant decrease in number and duration of analgesic narcotic therapies was observed. Other studies reported partial improvement; however, these studies often did not have a direct improvement on the specified clinical outcome [30,34].

We also examined the effect of model source (i.e., custom versus “off-the-shelf” model) on clinical outcomes. Overall, there was a trend of custom predictive models being associated with greater likelihood of improved clinical outcomes. Eleven of the studies that implemented custom models and one of the studies that implemented “off-the-shelf” models did not report the effects of model implementation on clinical outcomes. Of the 19 studies that implemented custom models and evaluated clinical outcomes, 16 (84.2%) studies showed an improvement in clinical outcomes while 3 (15.8%) studies reported no improvement in outcomes. Of the 13 studies that implemented “off-the-shelf” models and evaluated clinical outcomes, 6 (46.2%) showed an improvement in clinical outcomes, while 7 (53.8%) reported no improvement in outcomes (Table 3).

Additionally, we classified the included studies by intended end users of the model. Several studies did not report evaluation of effects of model implementation on clinical outcomes (six studies with physicians as primary intended users, three studies with nurses as primary intended users, and three studies where intended users were other health care workers). Of the 22 studies that evaluated clinical outcomes when physicians were the primary intended users of the model, 15 (68.2%) showed an improvement in clinical outcomes. Of the eight studies that evaluated clinical outcomes when only nurses were the intended users of the model, five studies (62.5%) reported improved outcomes after model implementation (Table 4). Therefore, there did not appear to be substantial differences in effect on clinical outcomes based on types of end users intended for the model.

### 3.6. Quality Assessment

The greatest proportion of studies were pre–post studies (19/44, 43.2%), followed by prospective cohort or validation studies (12/44, 27.3%). Studies with higher levels of evidence such as randomized controlled trials (5/44, 11.4%) comprised the minority of studies. Qualitative assessments guided by criteria detailed in the Downs and Black checklist [25] revealed that the study quality ranged widely from “limited” to “strong” ratings, with the majority demonstrating sufficient internal validity. However, because pre–post studies can be affected by general temporal trends, and observational study designs are less generalizable (i.e., less external validity), and furthermore most studies lacked control groups, we rated the overall quality of evidence from the included studies as low to moderate strength. The risk of bias across studies was reduced by searching multiple databases and using diverse and exhaustive search terms. Although publication and reporting bias may still exist, the use of multiple databases and exhaustive search terms increased the number of available records and expanded the search across multiple disciplines. In addition, only half of the included studies reported improvements in clinical outcomes, which suggests there is likely not a strong publication bias toward representing only positive findings.

## 4. Discussion

### 4.1. Summary of Evidence and Key Findings

In this systematic review, we observed several trends in the current literature published about the clinical implementation of predictive models embedded in EHR systems. Although predictive modeling has surged in the last decade, there is a paucity of research describing the integration of predictive models into EHR systems and implementation of those models into clinical usage in real-world settings.

To emphasize our focus on clinical implementation, we analyzed several factors of predictive models to examine their effect on clinical outcome. For instance, our results suggest that implementing custom models was more likely to improve clinical outcomes than implementing “off-the-shelf” models without site-specific customization. Some authors attributed the success of their model to the custom fit for their specific institution and input and engagement from in-house providers in the creation of the model [38]. However, the contrast between custom models and “off-the-shelf” models in improving clinical outcomes may be due to custom models being developed and validated in response to specific clinical issues. While some studies implementing “off-the-shelf” models reported success in improving patient outcomes, such as time to antibiotic administration and hospital length of stay [56], other studies suggested that plugging in a previously validated model without custom modifications, such as the CHADS2VASC score, exacerbated common implementation challenges due to lack of custom fit for the institution [47]. These findings support prior studies that have expressed the importance of local validation and customization, not just for predictive models but for EHR and health information technology (IT) systems more broadly [20,71,72].

End user education and training and workflow integration were also common themes. This finding supports the important role of the end user in several previously published frameworks concerning the implementation of emerging technologies in predictive analytics and artificial intelligence [20,73,74]. Institutional investment in training is critical, as quality of training has been shown to significantly influence users’ satisfaction with EHRs and health IT systems [75]. Similarly, the included studies frequently emphasized workflow considerations, such as the discussion by Fossum et al. [28] on the additional burden imposed by CDS on nurses burdened with an already high workload. One key concern was workflow interruption, with alert fatigue being a key issue highlighted by several studies (detailed in Table 2). Alert fatigue is a pervasive issue in providing effective CDS, and future studies will need to examine potentially new ways of information presentation to mitigate alert fatigue and the risk of clinicians ignoring potentially important information arising from predictive models.

Besides elements centered on end users such as training, workflow integration, and alert fatigue, other considerations for implementation concerned higher-level organizational issues. For example, a heart failure readmissions model described by Amarasingham [33] was not activated on weekends or holidays, instead focusing on weekdays when follow-up interventions for high-risk patients could be coordinated by a heart failure case manager. This illustrates the need for adequate personnel/staffing beyond patient-facing clinicians alone to implement some of the relevant interventions downstream from the model. Several studies [66,68] also cited the importance of adhering to organizational preferences, achieving buy-in from health system leadership, and designating “on-the-ground” champions to facilitate adoption. These concepts emphasize the importance of considering predictive models within the context of health systems more broadly during implementation.

Overall, a significant portion of studies was comprised of study designs with low to moderate levels of evidence (e.g., pre–post studies), while study designs with high levels of evidence (e.g., randomized controlled trials) comprised the minority. The prevalence of pre–post studies may be due to the natural progression of development and validation studies to pre–post intervention studies, often leveraging data generated during routine clinical care. Additionally, there may be a lack of high-evidence level study designs due to the recent adoption of predictive models into EHR over the last decade. The lack of randomized controlled trials reflects the need for high quality studies to ensure that predictive models can effectively transition from development and validation to clinical implementation.

### 4.2. Gaps in the Literature and Opportunities for Future Investigation

This systematic review highlighted several gaps in existing literature that can serve as opportunities for future investigation.

First, clinical domains with a high disease burden in inpatient settings (e.g., sepsis, thrombotic disorders, readmissions) were the most well represented, while outpatient conditions were relatively underrepresented. One reason may be that outpatient models found during the search process often satisfied partial criteria, but not the full eligibility criteria (e.g., predictive models that were developed but not yet available or implemented in the EHR). This may derive from the longer periods of time associated with outpatient clinical outcomes compared to the time-limited nature of inpatient encounters, such that outcome ascertainment for outpatient clinical domains may be better represented in the future as more time elapses. Another reason may be the greater quantity of data available from inpatient settings due to higher frequency of assessments (e.g., multiple vital sign measurements, laboratory values, etc. in a single day), while outpatient data are more limited per visit and take a longer period of time to accumulate. Models predicting clinical outcomes in the outpatient settings are critically important given that outpatient conditions impose the greatest disease burden, and because the vast majority of health care is delivered in outpatient settings.

Second, over a quarter of the included studies did not assess clinical outcomes. Several authors indicated that studies are ongoing, with results pertaining to clinical outcomes still pending following implementation. Evaluating clinical impact would be the next natural step for these studies, which highlights the relative recency of implementing predictive models into the EHR.

Third, among the predictive models included in this review, very few used computational methods more advanced than linear or logistic regression to develop the model. Although the use of machine learning in the development and validation of predictive models is gaining traction in the field of biomedical informatics, there is still a gap in evaluation of these models in terms of clinical implementation and outcomes. Almost a third of studies that evaluated clinical outcomes showed no improvements, thus warranting a closer examination of barriers to implementation and/or adoption.

### 4.3. Limitations

Due to the heterogeneous clinical domains and patient populations, we did not conduct a meta-analysis for the included studies, and thus we were unable to quantitatively assess effects on specific clinical domains across studies. In addition, our results cannot be generalized to studies outside of our eligibility criteria (e.g., predictive models outside of EHR systems).

This review also did not report on the logistic aspects of predictive model implementation that were outside the scope of this review. For instance, we did not include formal evaluation of implementation costs. Costs were mentioned in only 4 (9.0%) of the included articles, in several cases only briefly without rigorous economic evaluations. The limited number of studies available in the database Business Source Complete suggests this is not a well-studied area. Our search strategy was also limited to only English language articles and thus may not have captured implementations in non-English speaking countries.

While conducting this review, we had expected to find a larger number of predictive models based on machine learning and artificial intelligence. However, after implementing our inclusion and exclusion criteria, there were very few predictive models using these advanced computational methods that had been implemented in real clinical settings. This may be due to the relatively recent development of machine learning-based models and thus would require several more years to produce trends in clinical outcomes following implementation.

## 5. Conclusions

Within the last decade, predictive models in EHR systems have become more common in response to a growing amount of available data. In this systematic review, we focused on whether the rise in development and validation of predictive models has led to effective clinical implementation and improved patient outcomes. We have highlighted several key findings related to implementation of predictive models and identified several promising areas for future investigation. The low to moderate levels of evidence represented in the current studies highlight an opportunity for future randomized control trials and cohort studies to improve generalizability. Through this systematic review, we hope to provide guiding trends and themes to direct future studies towards establishing best practices for implementing EHR-based predictive models.

## Supplementary Material

Supplemental Figure S1

Supplemental Table S1

## Figures and Tables

**Table 1.** Overview of included studies pertaining to predictive models embedded in electronic health record (EHR) systems implemented in clinical settings.

Author	Year	Location	Study Design	Sample Size	Clinical Outcome(s)
Maynard et al. [26]	2010	California, USA	Retrospective cohort	748	Venous thromboembolism
Novis et al. [27]	2010	Illinois, USA	Pre–post	400	Deep vein thrombosis
Fossum et al. [28]	2011	Norway	Quasi-experimental *	971	Pressure ulcers, malnutrition
Herasevich et al. [29]	2011	Minnesota, USA	Pre–post	1159	Ventilator-induced lung injury
Nelson et al. [30]	2011	Michigan, USA	Pre–post	33,460	Sepsis
Umscheid et al. [31]	2012	Pennsylvania, USA	Pre–post	223,062	Venous thromboembolism
Baillie et al. [32]	2013	Pennsylvania, USA	Pre–post	120,396	Readmission
Amarasingham et al. [33]	2013	Texas, USA	Pre–post	1726	Readmission
Litvin et al. [34]	2013	South Carolina, USA	Prospective cohort	38,983	Chronic kidney disease
Oh et al. [35]	2014	South Korea	Pre–post	1111	Delirium
Resetar et al. [36]	2014	Missouri, USA	Prospective cohort	3691	Ventilator-associated events
Amland et al. [37]	2015	Missouri, USA	Pre–post	45,046	Venous thromboembolism
Faerber et al. [38]	2015	New Hampshire, USA	Pre–post	297	Mortality
Hao et al. [39]	2015	Maine, USA	Prospective cohort	118,951	Readmission
Kharbanda et al. [40]	2015	Minnesota, USA	Prospective cohort	735	Hypertension
Lustig et al. [41]	2015	Canada	Prospective cohort	580	Venous thromboembolism
Umscheid et al. [42]	2015	Pennsylvania, USA	Pre–post	15,526	Sepsis, deterioration
Depinet et al. [43]	2016	Ohio, USA	Pre–post	1886	Appendicitis
Narayanan et al. [44]	2016	California, USA	Pre–post	103	Sepsis
Vinson et al. [45]	2016	California, USA	Pre–post	893	Pulmonary embolism
Aakre et al. [46]	2017	Minnesota and Florida, USA	Prospective cohort	242	Sepsis
Arts et al. [47]	2017	Netherlands	Randomized controlled trial	781	Stroke
Bookman et al. [48]	2017	Colorado, USA	Pre–post	120	Use of imaging
Jin et al. [49]	2017	South Korea	Case-control	1231	Pressure injury
Samal et al. [50]	2017	Massachusetts, USA	Prospective cohort	569,533	Kidney failure
Shimabukuro et al. [51]	2017	California, USA	Case-control	67	Sepsis
Chaturvedi et al. [52]	2018	Florida, USA	Prospective cohort	309	Anticoagulant therapy
Cherkin et al. [53]	2018	Washington, USA	Randomized controlled trial	4709	Physical function and pain
Ebinger et al. [54]	2018	Minnesota, USA	Prospective cohort	549	Complications, mortality, length of stay, and cost
Hebert et al. [55]	2018	Ohio, USA	Prospective cohort	129	Ventilator-associated events
Jung et al. [56]	2018	Ohio, USA	Pre–post	232	Sepsis, mortality
Kang et al. [57]	2018	South Korea	Case-control	8621	Medical errors
Karlsson et al. [58]	2018	Sweden	Randomized controlled trial	444,347	Anticoagulant therapy
Moon et al. [59]	2018	South Korea	Retrospective cohort	4303	Delirium
Ridgway et al. [60]	2018	Illinois, USA	Prospective cohort	180	HIV
Turrentine et al. [61]	2018	Virginia, USA	Pre–post	1864	Venous thromboembolism
Villa et al. [62]	2018	California, USA	Pre–post	33,032	Triage time
Vinson et al. [63]	2018	California, USA	Pre–post	881	Pulmonary embolism
Bedoya et al. [64]	2019	North Carolina, USA	Retrospective cohort	85,322	Deterioration
Brennan et al. [65]	2019	Florida, USA	Quasi-experimental *	20	Preoperative risk assessment
Ekstrom et al. [66]	2019	California and Upper Midwest, USA	Prospective cohort	Not stated	Appendicitis
Giannini et al. [67]	2019	Pennsylvania, USA	Randomized controlled trial	54,464	Sepsis
Khoong et al. [68]	2019	California, USA	Randomized controlled trial	524	Chronic kidney disease
Ogunwole et al. [69]	2019	Texas, USA	Pre–post	204	Readmission, Heart failure

* Quasi-experimental study design refers to other non-randomized clinical trials that did not qualify as pre–post studies.

**Table 2.** Classification and method of risk score presentation of studies that discussed alert fatigue in relation to implementation of predictive models within electronic health record systems.

Author	Interruptive vs. Non-Interruptive	Description of Risk Score Presentation	Quotation Regarding Alert Fatigue
Arts et al. [47]	Non-Interruptive	Floating notification window	“Too many alerts will tend to result in all alerts being ignored, a phenomenon known as ‘alert fatigue.’ Given the possible adverse effects of ‘alert fatigue’ and interruption, we considered the optimal interface to be one which minimized these effects.”
Bedoya et al. [64]	Interruptive	Best practice advisory (BPA) triggered requiring response from care nurse	“The majority of BPAs were ignored by care nurses. Furthermore, because nurses were ignoring the BPA, the logic in the background would cause the BPA to repeatedly fire on the same patient. This in turn created a large quantity of alerts that required no intervention by clinicians and led to alert fatigue in frontline nursing staff. Anecdotal feedback from nurses confirmed the constant burden of alerts repeatedly firing on individual patients. Furthermore, alert fatigue begets more alert fatigue and the downstream consequences of alert fatigue can include missed alerts, delay in treatment or diagnosis, or impaired decision-making when responding to future alerts.”
Depinet et al. [43]	Interruptive	Alert, data collection screen and feedback interface	“The firing of the CDS tool each time there was a chief complaint related to appendicitis may have led to alert fatigue. Overall, more work is needed to introduce a culture of standardized care in which such a decision support tool could work optimally.”
Herasevich et al. [29]	Interruptive	Bedside alert via text paging	“Because the majority of patients are treated with appropriate ventilator settings, unnecessary interruptions with new alert paradigms could have a detrimental effect on performance. It is therefore critical to incorporate contextual smiddle rules within decision support systems to prevent false positive alerts. Interruptions are often seen as distracting or sometimes devastating elements that need to be minimized or eliminated.”
Jin et al. [49]	Non-Interruptive	Display on nursing record screen	“Most computerized risk assessment tools require that nurses measure each score for each item in the scale. Thus, risk assessment scores are obtained only if all item scores are entered into the EHR system. Hence, as reported in a previous study, nurses have experienced work overload and fatigue and expressed their preference to use the paper charts. In addition, nurses felt a lot of time pressure.”
Kharbanda et al. [40]	Interruptive	Alert and dashboard display	“Four of eight (50 percent) rooming staff respondents reported that alerts to remeasure a BP [blood pressure] ‘sometimes’ interfered with their workflow, and the remaining responded that the alerts ‘rarely interfered.’”
Oh et al. [35]	Non-Interruptive	Pop-up window displayed on primary electronic medical record screen	“Most of the nurses did not recognize the urgent need for delirium care and did not consider it part of their regular routine. Therefore, nurses considered the additional care indicated by the system as extra work.”
Shimabukuro et al. [51]	Interruptive	Alert via phone call to charge nurse	“Systems that use these scores deliver many false alarms, which could impact a clinician’s willingness to use the sepsis classification tool.”

**Table 3.** Distribution of studies regarding source of predictive model and improvement in clinical outcomes after implementation. Only studies that reported evaluations of effects of model implementation on clinical outcomes are included in the table.

	Custom Model (*n* = 19)	“Off-the-Shelf” Model (*n* = 13)
Improved clinical outcomes	16 (84.2%)	6 (46.2%)
No improvements in outcomes	3 (15.8%)	7 (53.8%)

**Table 4.** Distribution of studies regarding intended users of EHR-based predictive models and improvement in clinical outcomes after implementation. Only studies that reported evaluations of effects of model implementation on clinical outcomes are included in the table.

	Physicians as Primary Intended Users (*n* = 22)	Nurses as Primary Intended Users (*n* = 8)	Other Intended Users^1^ (*n* = 2)
Improved clinical outcomes	15 (68.2%)	5 (62.5%)	2 (100%)
No improvements in outcomes	7 (31.8%)	3 (37.5%)	0 (0%)

1 Other intended users include all cases where physicians and/or nurses were not the intended primary end users, including but not limited to respiratory therapists, rapid response coordinators, counselors, or unreported users.

## Appendix A Detailed PubMed Search Strategy

(((((((((((((((((((electronic data processing[MeSH Terms]) OR health plan implementation[MeSH Terms]) OR implementation science[MeSH Terms]) OR pragmatic clinical trials as topic[MeSH Terms]) OR implementation*[Title/Abstract]) OR pragmatic clinical trial*[Title/Abstract]) OR pragmatic trial*[Title/Abstract]) OR randomized clinical trial*[Title/Abstract]) OR real world[Title/Abstract]) OR real-world[Title/Abstract]) OR real time[Title/Abstract]) OR real-time[Title/Abstract]) OR applied clinical trial*[Title/Abstract]) OR practical clinical trial*[Title/Abstract]) OR bedside technology[Title/Abstract]) OR bedside comput*[Title/Abstract]) OR prototype*[Title/Abstract])) AND (((((((((((((((((((((((((((((((((((((((((((((((algorithm[MeSH Terms]) OR artificial intelligence[MeSH Terms]) OR cluster analysis[MeSH Terms]) OR deep learning[MeSH Terms]) OR logistic model[MeSH Terms]) OR machine learning[MeSH Terms]) OR unsupervised machine learning[MeSH Terms]) OR supervised machine learning[MeSH Terms]) OR clinical decision support systems[MeSH Terms]) OR decision support systems, clinical[MeSH Terms]) OR algorithm*[Title/Abstract]) OR artificial intelligence*[Title/Abstract]) OR deep learning*[Title/Abstract]) OR logistic model*[Title/Abstract]) OR machine learning*[Title/Abstract]) OR clinical decision support*[Title/Abstract]) OR medical decision support*[Title/Abstract]) OR adaptive health system*[Title/Abstract]) OR risk prediction*[Title/Abstract]) OR learning health system*[Title/Abstract]) OR digital phenotyp*[Title/Abstract]) OR outcome prediction*[Title/Abstract]) OR phenotyping algorithm*[Title/Abstract]) OR prediction model*[Title/Abstract]) OR predictive model*[Title/Abstract]) OR risk flag*[Title/Abstract]) OR risk score*[Title/Abstract]) OR risk stratif*[Title/Abstract]) OR risk assessment[Title/Abstract]) OR risk classif*[Title/Abstract]) OR semi-supervised learning[Title/Abstract]) OR statistical model*[Title/Abstract]) OR probabilistic model*[Title/Abstract]) OR predictive value of tests[Title/Abstract]) OR probabilistic learning[Title/Abstract]) OR probability learning[Title/Abstract])OR neural network*[Title/Abstract])OR clinical prediction rule*[Title/Abstract]) OR clinical prediction tool*[Title/Abstract]) OR clinical prediction score*[Title/Abstract]) OR machine intelligence[Title/Abstract]) OR AI[Title/Abstract]) OR prognostic tool*[Title/Abstract]) OR prediction algorithm*[Title/Abstract]) OR predictive algorithm*[Title/Abstract]))) AND (((((((((((((((electronic health record[MeSH Terms]) OR computerized medical record[MeSH Terms]) OR computerized medical record system[MeSH Terms]) OR medical order entry systems[MeSH Terms]) OR electronic health record*[Title/Abstract]) OR computerized medical record*[Title/Abstract]) OR electronic medical record*[Title/Abstract]) OR digital medical record*[Title/Abstract]) OR digitized medical record*[Title/Abstract]) OR digital health[Title/Abstract]) OR computerized patient record*[Title/Abstract]) OR electronic patient record*[Title/Abstract]) OR automated patient record*[Title/Abstract]) OR digital patient record*[Title/Abstract]) OR digitized patient record*[Title/Abstract])
