# Supplementary figures and images for: Clinical Implementation of Predictive Models Embedded within Electronic Health Record Systems: A Systematic Review
Source: Informatics (MDPI). Author manuscript; Available in PMC 2020 Dec 2. (PMC7710328; doi:10.3390/informatics7030025)

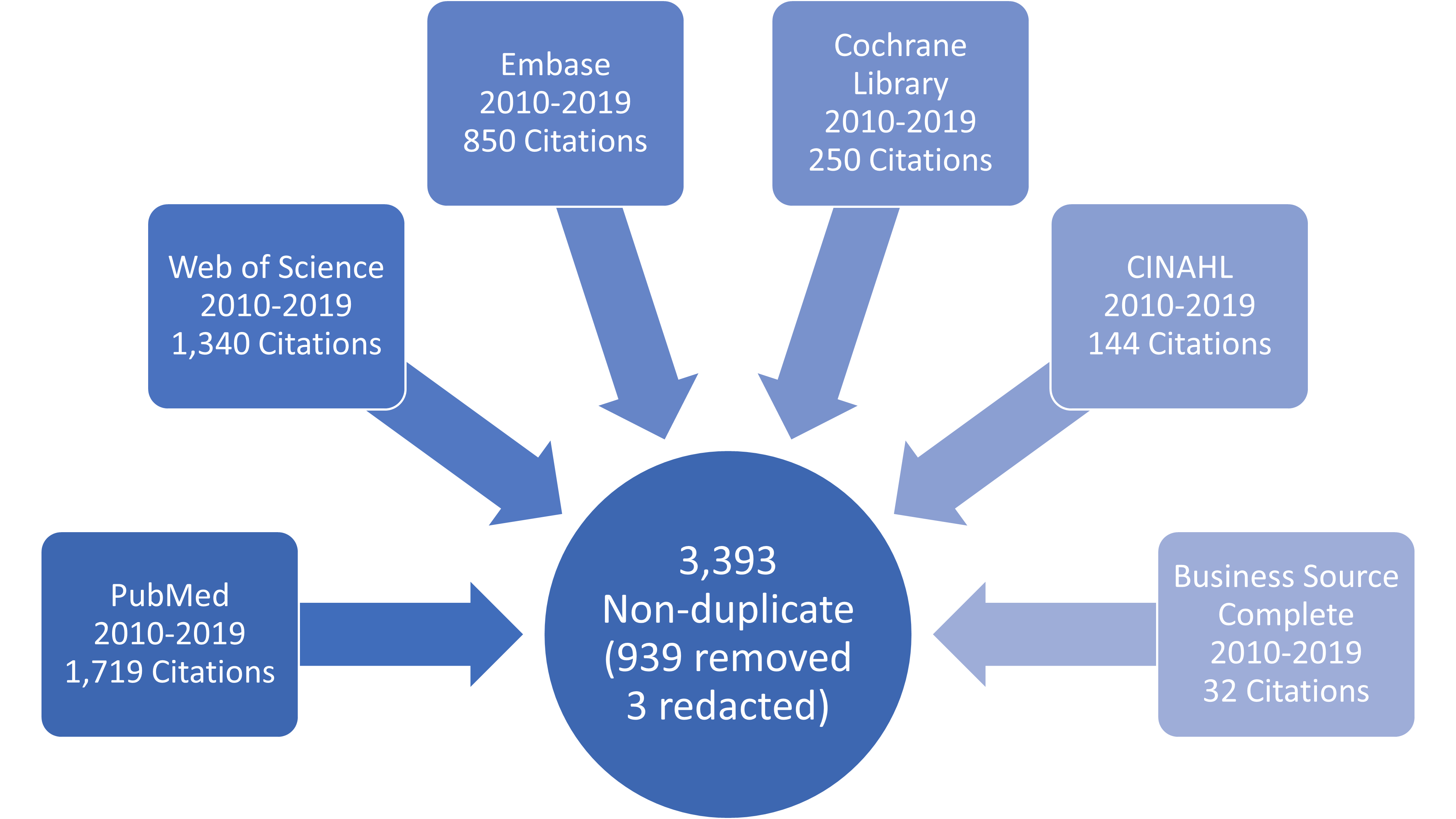

Supplement: Supplemental Figure S1 [file NIHMS1644136-supplement-Supplemental_Figure_S1.tif]
